# Clinical significance of clonal hematopoiesis in the interpretation of blood liquid biopsy

**DOI:** 10.1002/1878-0261.12727

**Published:** 2020-06-08

**Authors:** Hiu Ting Chan, Satoshi Nagayama, Yoon Ming Chin, Masumi Otaki, Rie Hayashi, Kazuma Kiyotani, Yosuke Fukunaga, Masashi Ueno, Yusuke Nakamura, Siew‐Kee Low

**Affiliations:** ^1^ Cancer Precision Medicine Center Japanese Foundation for Cancer Research Tokyo Japan; ^2^ Department of Gastroenterological and Surgery Cancer Institute Hospital Japanese Foundation for Cancer Research Tokyo Japan; ^3^ Cancer Precision Medicine, Inc Kawasaki Japan

**Keywords:** circulating tumor DNA, clonal hematopoiesis, colorectal cancer, liquid biopsy, next‐generation sequencing

## Abstract

As the use of next‐generation sequencing (NGS) for plasma cell‐free DNA (cfDNA) continues to expand in clinical settings, accurate identification of circulating tumor DNA mutations is important to validate its use in the clinical management for cancer patients. Here, we aimed to characterize mutations including clonal hematopoiesis (CH)‐related mutations in plasma cfDNA and tumor tissues using the same ultradeep NGS assay and evaluate the clinical significance of CH‐related mutations on the interpretation of liquid biopsy results. Ultradeep targeted NGS using Oncomine Pan‐Cancer Panel was performed on matched surgically resected tumor tissues, peripheral blood cells (PBCs), and 120 plasma cfDNA samples from 38 colorectal cancer patients. The clinical significance of the CH‐related mutations in plasma cfDNA was evaluated by longitudinal monitoring of the postoperative plasma samples. Among the 38 patients, 74 nonsynonymous mutations were identified from tumor tissues and 64 mutations from the preoperative plasma samples. Eleven (17%) of the 64 mutations identified in plasma cfDNA were also detected in PBC DNA and were identified to be CH‐related mutations. Overall, 11 of 38 (29%) patients in this cohort harbored at least one CH‐related mutation in plasma cfDNA. These CH‐related mutations were continuously detected in subsequent postoperative plasma samples from three patients which could be misinterpreted as the presence of residual disease or as lack of treatment response. Our results indicated that it is essential to integrate the mutational information of PBCs to differentiate tumor‐derived from CH‐related mutations in liquid biopsy analysis. This would prevent the misinterpretation of results to avoid misinformed clinical management for cancer patients.

## Graphical abstract

Identification of circulating tumor DNA mutations through next‐generation sequencing (NGS) of plasma samples has important clinical applications. However, a substantial fraction of mutations detected shares features of mutations related with clonal hematopoiesis. Paired plasma and peripheral blood cell sequencing should be standard practice for NGS analysis of liquid biopsies to avoid misinterpretation of results and misinformed clinical management of cancer patients.

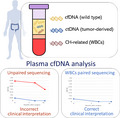

**Abbreviations**
cfDNA: cell‐free DNA
CH: clonal hematopoiesis
NGS: next‐generation sequencing
PBC: peripheral blood cell
SNV: single nucleotide variant
VAF: variant allele frequency

## 1 Introduction

The development of next‐generation sequencing (NGS) technology has allowed in‐depth characterization of tumor genomes and resulted in a better understanding of the molecular aberrations across different cancer types (Zehir *et al*., 2017). However, the difficulty to obtain tumor tissues at multiple time points and intra‐ and intertumoral heterogeneity of individual cancer patients limited the ability to achieve real‐time and accurate characterization of tumors (Heitzer *et al*., 2019). In this regard, the minimally invasive nature of liquid biopsy overcomes these issues. Recent studies have shown the potential of genomic analysis of plasma cell‐free DNA (cfDNA) to facilitate early cancer detection (Bettegowda *et al*., 2014; Cree *et al*., 2017; Lam *et al*., 2018), residual disease detection (Chaudhuri *et al*., 2017; Tie *et al*., 2016), and disease and treatment monitoring (Demuth *et al*., 2018; Long‐Mira *et al*., 2018; Váraljai *et al*., 2019) that could lead to the era of precision oncology.

The discordance of mutations detected from tumor tissues and plasma cfDNA reported in previous studies is one of the major challenges for the application of liquid biopsy (Bolivar *et al*., 2019; Chae *et al*., 2016; Wyatt *et al*., 2017). Several reasons may explain this discordance. Firstly, early‐stage cancers with a small‐size tumor may not release sufficient amount of DNA to be detected by plasma liquid biopsy leading to the discordance observed (Fiala and Diamandis, 2018). Secondly, cfDNA genomic analysis is more likely to represent the entire tumor while sequencing tumor tissues or biopsy samples is unlikely to represent intra‐ and intertumoral heterogeneity (Nelson *et al*., 2018; Richman *et al*., 2011). Nonstandardized NGS assays, platforms, and methodologies across different sample sets may also contribute to the discordance observed between tumor tissue DNA and plasma cfDNA (Neumann *et al*., 2018). The complex composition of plasma cfDNA, which contains a mixture of mutations derived from germline DNA, clonally expanded hematopoietic cells, and malignant cells may also contribute as a source of discordance (Merker *et al*., 2018; Wan *et al*., 2017). Furthermore, whole‐genome array analyses of cfDNA have demonstrated that most cfDNAs were of hematopoietic origins, suggesting genomic aberrations from hematopoietic cells may also be detected from plasma (Kustanovich *et al*., 2019). Such observations have been reported in several studies where clonal hematopoiesis (CH)‐related mutations, which are somatic mutations derived from nonmalignant hematopoietic cells, can also be detected in tumor tissue DNA or plasma cfDNA (Coombs *et al*., 2018; Hu *et al*., 2018; Ptashkin *et al*., 2018). A recent study conducted by Razavi *et al*. (2019) reported that up to 50% of cfDNA mutations detected from cancer patients and 80% from healthy controls had features consistent with CH. These observations highlighted the potential significance of CH‐related mutations in cfDNA genomic analysis and their impact on the annotation of the variants detected from blood liquid biopsy. Understanding the contribution of CH‐related mutations to the somatic mosaicism in cfDNA and their clinical implications is important for the accurate clinical application of liquid biopsy (van der Leest and Schuuring, 2020). In this study, we validated the detection of CH‐related mutations in plasma cfDNA and tumor tissue DNA using the same NGS platform and evaluated the impact of screening CH‐related variants on the interpretation of liquid biopsy in clinical settings.

## 2 Methods

### 2.1 Patient cohort and sample collection

Matched tumor tissues, peripheral blood cells (PBCs), and multiple time‐point cfDNA samples were collected from 38 patients that were diagnosed with colorectal adenocarcinoma from 2018 at the Cancer Institute Hospital, Japanese Foundation for Cancer Research, Tokyo, Japan. All eligible patients included in this study were pathologically confirmed as stage I to IV colorectal adenocarcinoma and were not subjected to chemotherapy or radiation therapy prior to tumor resection. Tumor tissues and peripheral blood samples were collected at the time of surgery. One of the three stage IV patients received simultaneous resection of the primary tumor and solitary liver metastatic lesion, and the remaining two stage IV patients underwent the resection of the solitary peritoneal dissemination along with the primary tumor. Therefore, there were no macroscopic residual tumors in all patients including stage IV patients. Surgically resected tumor tissues were stored in −80 °C until DNA extraction. Fourteen millilitre of peripheral blood was collected into EDTA‐2Na tubes (Terumo, Tokyo, Japan) at each time‐point and was centrifuged at 2000 ***g*** at 4 °C for 10 min within 30 min of collection. The obtained plasma samples were further centrifuged at 16 000 ***g*** at 4 °C for 10 min to remove cell debris. The separated plasma and buffy coat were stored at −80 °C until nucleic acid extraction. Multiple blood samples were also collected postoperatively for longitudinal monitoring. The clinical and pathological information was obtained from the pathology reports and the electronic medical record for each patient. Written informed consent was obtained from each patient at the time of sample collection. The study methodologies conformed to the standards set by the Declaration of Helsinki and were approved by the ethics committee in Japanese Foundation for Cancer Research (IRB‐2013‐1093).

### 2.2 DNA/RNA extraction

A total of 120 pre‐ and postoperative plasma samples were collected from 38 patients, and the cell‐free total nucleic acid, which includes both DNA and RNA, was extracted using the MagMAX Cell‐Free Total Nucleic Acid Isolation Kit (Applied Biosystems, Austin, TX, USA) according to the manufacturer's protocol. DNA was extracted from frozen tumor tissues using the AllPrep DNA Mini Kit (Qiagen, Hilden, Germany) according to the manufacturer's protocol. Frozen buffy coat samples were treated with the Red Blood Cell Lysis Buffer (BioLegend, San Diego, CA, USA), and DNA from a total of 2 × 10^6^ white blood cells was extracted using the AllPrep DNA Mini Kit. Extracted cell‐free total nucleic acid and genomic DNA (both buffy coat and tumor tissues) were quantified using Qubit DNA HS Assay Kit and Qubit DNA Broad Range Assay Kit (Life Technologies, Eugene, OR, USA), respectively. Quality of the extracted DNA was assessed using the TapeStation System (Agilent, Clara, CA, USA) via either Genomic DNA ScreenTape (tumor and PBC DNA) or High Sensitivity D5000 ScreenTape (cell‐free total nucleic acid) (Agilent).

### 2.3 Library preparation and targeted next‐generation sequencing

Library preparation for each sample was performed using the amplicon‐based Oncomine Pan‐Cancer Cell‐Free Assay following the manufacturer's protocol (Life Technologies, Frederick, MD, USA) with an input of 9–20 ng of cell‐free total nucleic acid. The panel includes a single pool of 272 multiplex PCR primers that cover 969 hotspot single nucleotide variants (SNVs), 12 copy number variations, and 12 gene arrangements across 53 genes. In brief, RNA from the cell‐free total nucleic acid input was reversely transcribed to cDNA and the total DNA was amplified using the panel primers that were tagged with unique molecular barcodes. Tag Sequencing Barcode (Life Technologies, Carlsbad, CA, USA) was utilized for multiplexing barcoded samples for sequencing. Lastly, the barcoded libraries were purified with size‐selection AMPure XP beads (Beckman Coulter, Brea, CA, USA) and quantified using the Ion TaqMan Quantification Kit (Thermo Fisher Scientific, Vilnius, Lithuania). Libraries were multiplexed for templating on the Ion Chef Instrument and subsequently sequenced on the Ion S5 Prime System using the Ion 540 or 550 Chip Kit. Both tumor and PBC DNA were mechanically sheared to 150 bps to mimic the average DNA length of cfDNA before library construction. Similar sequencing methodology was applied for DNA extracted from tumor tissue and buffy coat with an input of 20 ng.

### 2.4 Sequencing data analysis and statistical analysis

The alignment of sequencing raw data was performed by the torrent suite Software version 5.10.1 (Thermo Fisher Scientific) using TMAP with the default analysis parameters. The BAM files generated were then further analyzed by the customizable workflow from the ion reporter software (Thermo Fisher Scientific) for variant calling. Oncomine TagSeq Pan‐Cancer Liquid Biopsy w2.1 version 5.10 workflow was used. A minimum of three reads with the same molecular barcode was required to form a functional family, and a minimum of two variants supporting functional families was required to make SNP, MNP, and INDEL callings. Statistical analysis was performed in graphpad prism (version 8.1.1; GraphPad Software, San Diego, CA, USA). Mutations detected from PBCs were categorized as CH‐related mutations in this study. Nonparametric Mann–Whitney test was performed to compare the mean of variant allele frequencies (VAF) in different groups. All statistical tests were considered significant when *P*‐value was < 0.05.

## 3 Results

### 3.1 Patient characteristics and mutations detected from tumor tissue and plasma

A total of 38 colorectal adenocarcinoma patients were enrolled in this study with a median patient age at the initial sample collection point of 64.5 years old (42–88 years old). Among them, 20 were diagnosed with stage I or II and the remaining 18 patients were diagnosed at stage III or IV (Table S1). One of the three stage IV patients received simultaneous resection of the primary tumor and solitary liver metastatic lesion, and the remaining two stage IV patients underwent the resection of the solitary peritoneal dissemination along with the primary tumor. Therefore, there were no macroscopic residual tumors in all patients including stage IV patients. The median of extracted cell‐free total nucleic acid concentration was 4.55 ng·mL^−1^ (range: 2.23–28.54) which allowed a DNA/RNA input of 9–20 ng for ultradeep targeted NGS. The median sequencing depths were 18453x, 48622x, and 46202x, for tumor tissues, PBCs, and cfDNA, respectively. The median molecular depths were 2048x, 2581x, and 4004x, for tumor tissues, PBCs, and cfDNA, respectively (Table S2). Mutation analysis of tumor tissue DNA identified 74 nonsynonymous mutations in 10 genes, and 33 of the 38 patients carried at least one mutation (Table S3, Fig. S1). The most commonly mutated genes were *TP53* (46%), *KRAS* (19%), *PIK3CA* (11%), and *APC* (10%) (Fig. S1). Sixty‐four mutations were detected from the plasma cfDNA, and 31 patients carried at least one mutation (Table S4, Fig. S1). Similar to the mutations detected from tumor tissues, the most commonly altered genes identified from plasma cfDNA were *TP53* (55%), *KRAS* (14%), and *APC* (9%) (Fig. S1).

### 3.2 Concordance of mutations detected in tumor tissues and plasma cfDNA

**Fig. 1:**
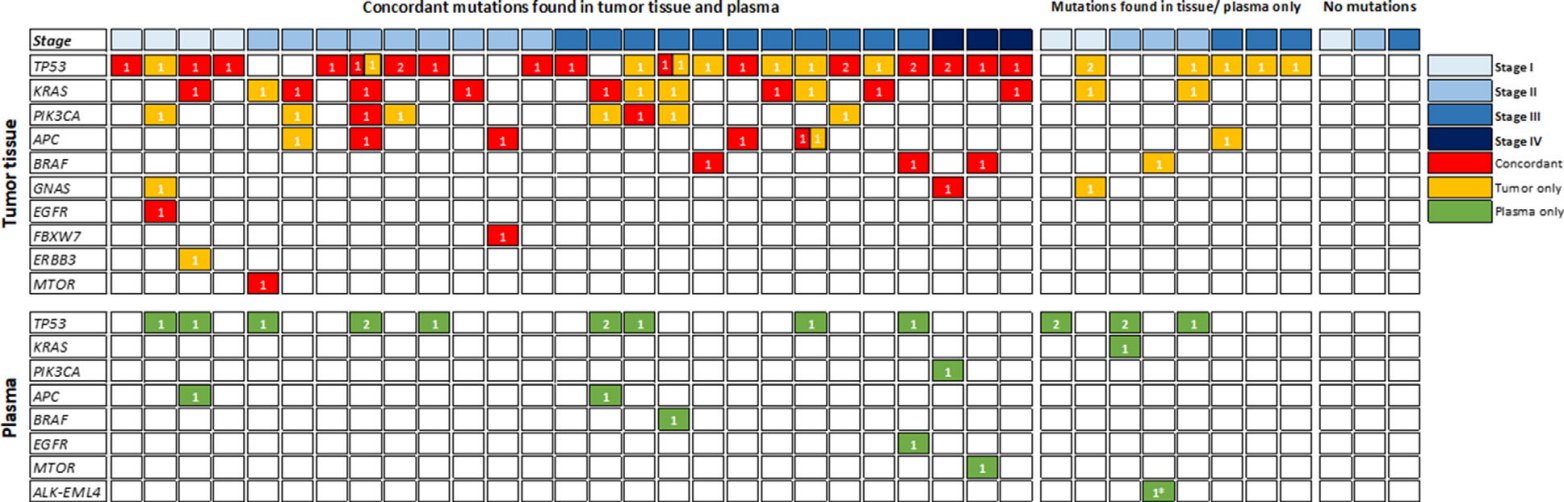
Summary of mutations identified from tumor tissues and preoperative plasma of the study cohort. A total of 74 nonsynonymous mutations were identified from tumor tissues, and 64 mutations were identified from preoperative plasma samples. No mutations from either tumor tissues or plasma were detected from three patients (*n* = 38).

**Fig. 2:**
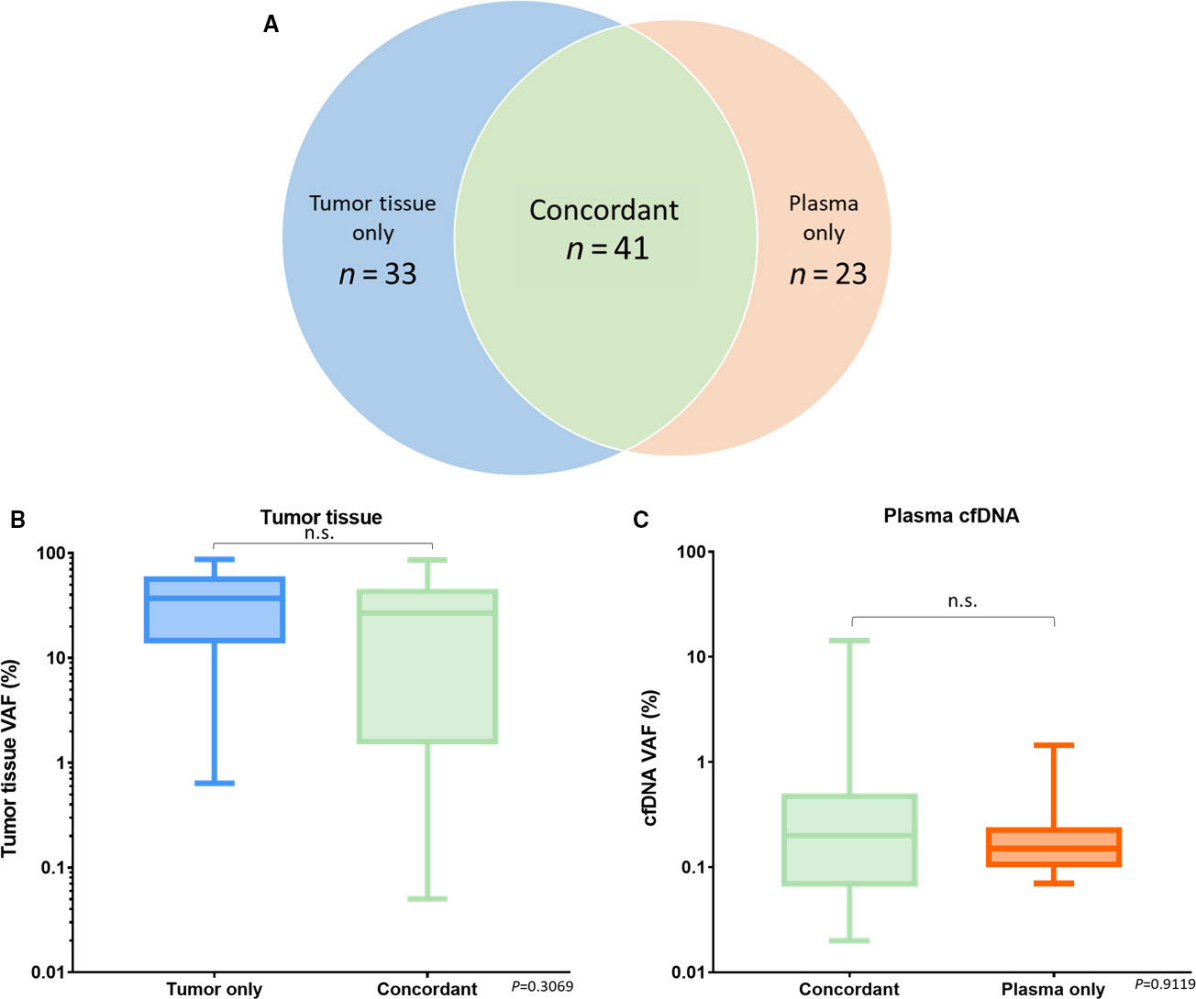
Concordance of mutations detected from tumor tissue DNA and plasma cfDNA. (A) Number of mutations identified from tumor tissue, preoperative plasma, or both sample sources. (B) The VAF of the mutations detected from tumor tissues (*n* = 74). The mean of the VAF of the concordant mutations detected from tumor tissues and plasma was not significantly different to the mean of the VAF of the mutations detected exclusively to tumor tissue (*P* = 0.3069). (C) The VAF of the mutations detected from plasma (*n* = 64). The mean of the VAF of the concordant mutations detected from tumor tissues and plasma was not significantly different to the mean of the VAF of the mutations detected exclusively to plasma (*P* = 0.9119). Nonparametric Mann–Whitney test was performed to compare the mean of VAF in different groups.

The summary of the mutations identified in tumor tissue DNA and plasma cfDNA is presented in Fig. 1. At least one common mutation in both tumor DNA and plasma cfDNA was detected in 71% (27/38) of the recruited patients. No mutation was detected in either tumor DNA or plasma cfDNA of three patients (Fig. 1). A total of 41 (55.4%) of the 74 mutations identified in tumor DNA were also identified in the matched plasma samples (Fig. 2A). No statistically significant differences were observed between the VAF of the concordant mutations and the mutations detected exclusively to tumor tissue or plasma (Fig. 2B,C).

### 3.3 Clonal hematopoiesis‐related mutations in tumor tissue and plasma samples

**Fig. 3:**
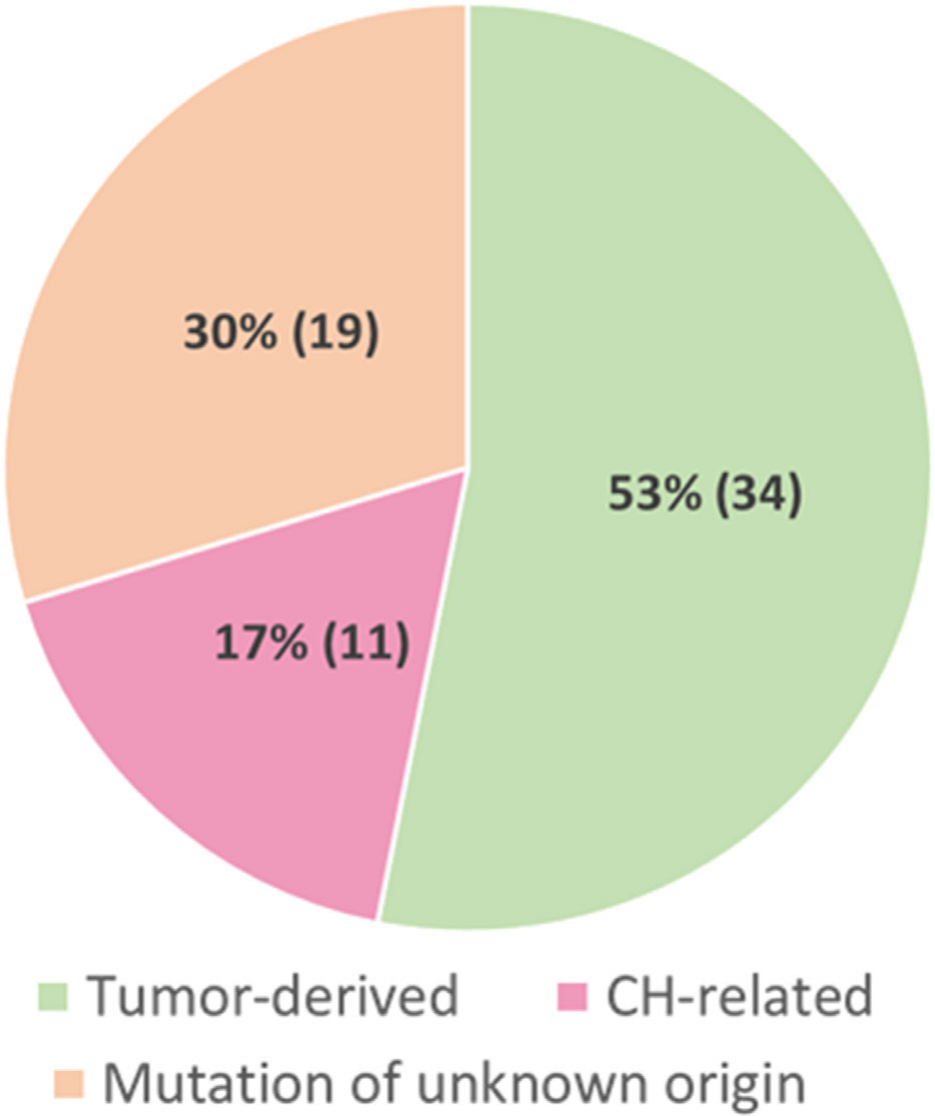
Clonal hematopoiesis‐related mutations detected from plasma. The distribution of somatic variants detected from preoperative plasma (*n* = 64). A total of 53% of mutations were concordantly detected from tumor tissue and confirmed to be tumor‐derived and 17% of mutations detected from plasma were also detected from PBCs, suggesting their hematopoietic origin. Up to 30% of mutations detected were plasma exclusive with unknown origin. The numbers in brackets represent the number of mutations from each source.

**Fig. 4:**
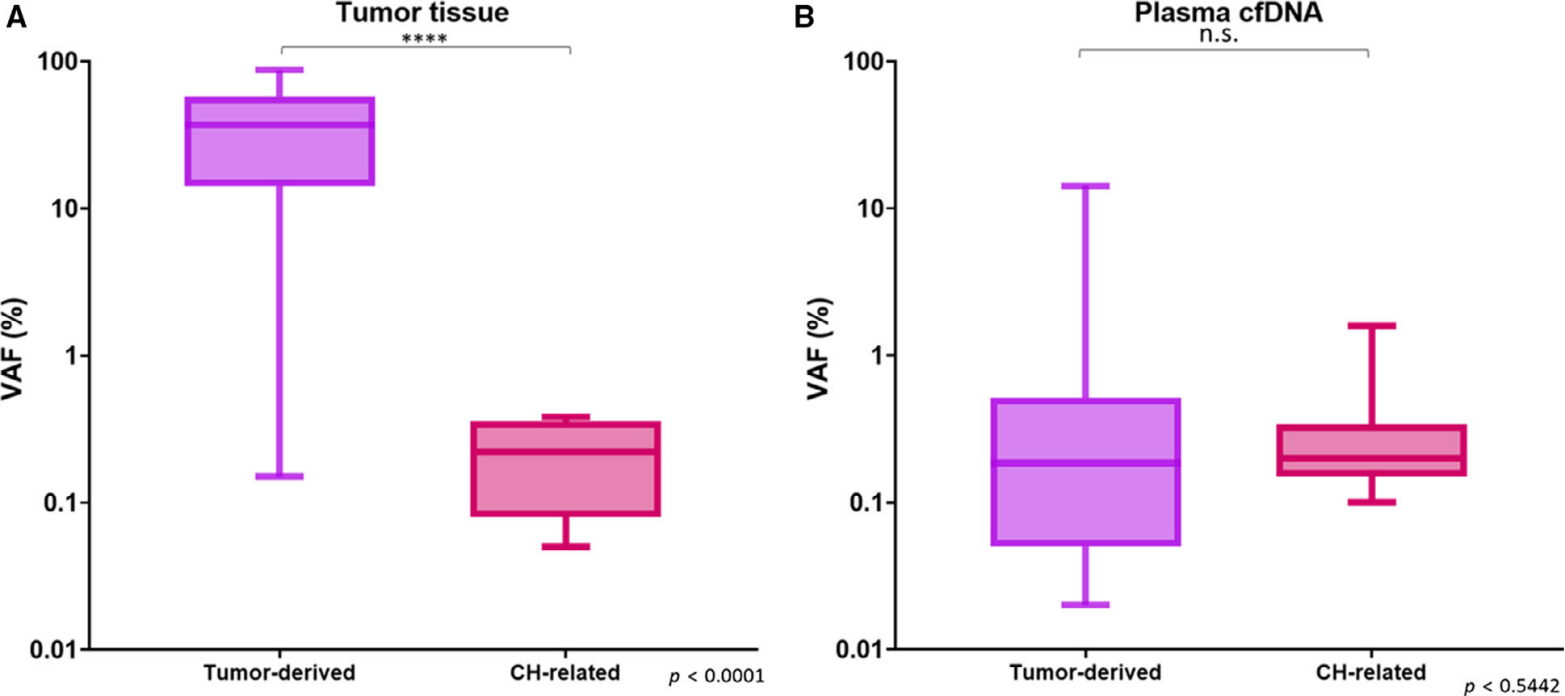
Variant allele frequency distribution of tumor‐derived mutations and CH‐related mutations from tumor tissue and plasma. (A) The VAF of the mutations detected from tumor tissues (*n* = 74). The mean of the VAF of the CH‐related mutations detected from tumor tissues was significantly different to the mean of the VAF of the tumor‐derived mutations detected from tumor tissues (*P* < 0.0001). (B) The VAF of the tumor‐derived and CH‐related mutations detected from preoperative plasma (*n* = 45). Mutations with unknown origin were excluded for this analysis. The mean of the VAF of the CH‐related mutations detected from plasma was not significantly different to the mean of the VAF of the tumor‐derived mutations detected from plasma (*P* = 0.5442). Nonparametric Mann–Whitney test was performed to compare the mean of VAF in different groups. ****P < 0.0001

Ultradeep sequencing was performed for the corresponding PBC samples of the 38 patients to characterize the sources of the cfDNA mutations that were detected from plasma. Eleven mutations detected from plasma were also detected from the PBCs suggesting their hematopoietic origin (Table 1). Furthermore, an increasing trend in the prevalence of PBC variants with age was also observed in this study cohort where up to 80% of patients above the age of 80 were detected with at least one somatic mutation from PBCs (Fig. S2). These observations were consistent with the features of CH‐related mutations. Based on these observations and interpretations, paired sequencing of cfDNA and PBCs suggests that 17% (11/64) of mutations detected from plasma were likely to be CH‐related (Fig. 3). Ten of these 11 mutations were identified in the *TP53* gene, and one was found in the *GNAS* gene. Our data indicated 29% (11 out of 38) of the patients in the cohort carried at least one CH‐related mutation, which were detectable by plasma liquid biopsy analysis using ultradeep sequencing method. Notably, among the 11 CH‐related mutations, seven mutations were also detected from tumor tissues (Table 1). The mean VAF of the tumor‐derived mutations detected from tumor tissue was significantly higher than that of the CH‐related mutations (*P* < 0.0001; Fig. 4A). In contrast, no statistical differences were observed in the VAF of the tumor‐derived mutations and the CH‐related mutation detected in the plasma samples (Fig. 4B).

**Table 1:** Summary of mutations detected from PBCs and their corresponding VAF detected from tumor tissue and plasma. N.D, not detected.

Patient	Locus	Genotype	Type	Genes	Amino acid change	VAF (%)	
						PBCs	Tumor tissue	Plasma
C02	chr17:7577568	C/G	SNV	*TP53*	p.C238S	1.40	0.36	1.58
C08	chr17:7577535	C/A	SNV	*TP53*	p.R249M	0.11	N.D	0.13
C11	chr17:7577120	C/T	SNV	*TP53*	p.R273H	0.08	N.D	0.19
C12	chr20:57484420	C/T	SNV	*GNAS*	p.R201C	0.10	0.05	0.32
C14	chr17:7578536	T/G	SNV	*TP53*	p.K132Q	0.23	N.D	0.18
C23	chr17:7577094	G/A	SNV	*TP53*	p.R282W	0.08	0.11	0.20
C25	chr17:7578457	C/T	SNV	*TP53*	p.R158H	0.06	0.35	0.15
C26	chr17:7578263	G/A	SNV	*TP53*	p.R196*	0.37	N.D	0.28
C27	chr17:7577570	C/T	SNV	*TP53*	p.M237I	0.05	0.08	0.34
C37	chr17:7577121	G/A	SNV	*TP53*	p.R273C	0.08	0.38	0.35
C44	chr17:7578403	C/T	SNV	*TP53*	p.C176Y	0.06	0.22	0.10

### 3.4 Clinical significance of accurate circulating tumor DNA mutation detection from plasma

**Fig. 5:**
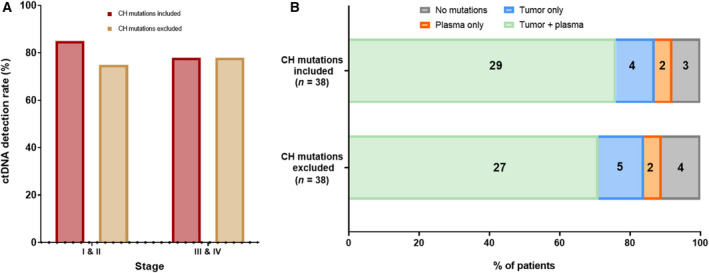
Mutation detection rate from preoperative plasma and tumor tissue before and after the exclusion of CH‐related mutations. (A) Percentage of patients detected with at least one mutation from plasma. The mutation detection rate from plasma for stage I and stage II patients was reduced from 85% to 75% after the exclusion of CH‐related mutations that were detected from plasma. No differences were observed for stage III and IV patients. (B) Percentage of patients harbored at least one mutation from plasma or tumor tissue for longitudinal monitoring before and after the exclusion of CH‐related mutations.

**Fig. 6:**
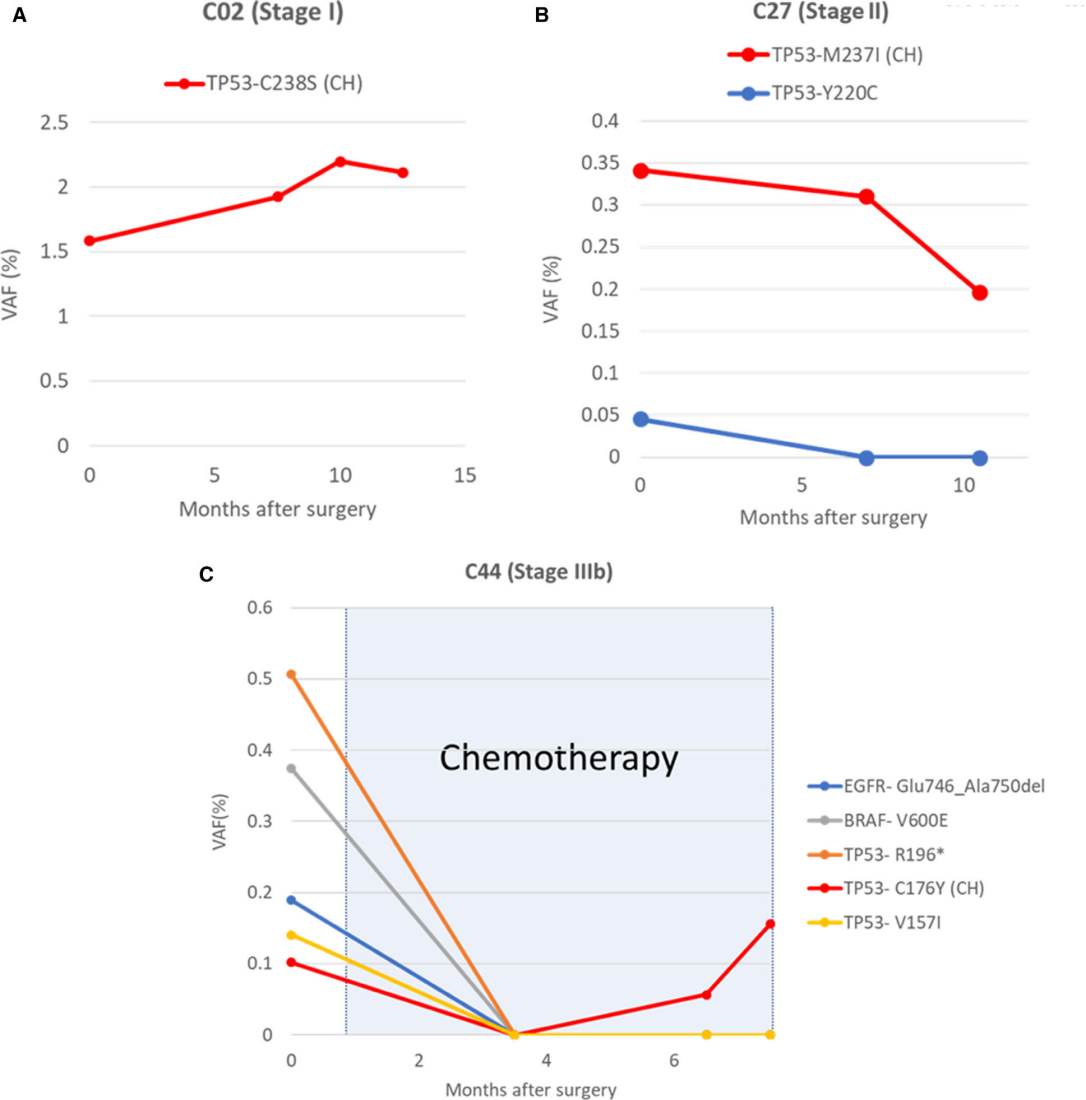
Misidentification of CH‐related mutations as tumor‐derived mutations in unpaired plasma cfDNA ultradeep NGS sequencing. (A) Patient 2, stage I, was detected with one CH‐related mutation (*TP53*‐C238S) from both preoperative plasma and tumor tissue. The same mutation was detected in all postoperative plasma samples (7.5, 10, and 12.5 months after surgery) at a comparable VAF. (B) Patient 27, stage II, was detected with two mutations in the preoperative plasma sample. Mutation *TP53*‐M237I was also detected from PBCs and confirmed to be CH‐related. The CH‐related mutation was also detected in the postoperative plasma samples, while the tumor‐derived mutation, *TP53*‐Y220C, was no longer detected after tumor resection. (C) Patient 44, stage IIIb, was initiated with chemotherapy 1.5 months after primary tumor resection. Four tumor‐derived mutations were detected from preoperatively plasma and were no longer detected after surgery. One CH‐related mutation was detected from both preoperative and postoperative plasma samples.

After the exclusion of 11 CH‐related mutations, the detection rate of circulating tumor DNA (ctDNA) mutation remained unchanged in stage III or IV colorectal cancer patients (78%) as all patients were detected with more than one mutation in the plasma cfDNA. In contrast, the ctDNA detection rate of stage I and II patients was reduced from 85% to 75% after the removal of CH‐related mutations (Fig. 5A). Paired PBC sequencing identified one patient, who was only detected with one CH‐related mutation and no tumor‐derived mutations detected from tumor tissues or plasma (Fig. 5B). This gives a total of 89.5% of patients (34/38) to be detected with at least one mutation from tumor tissue or plasma that could be monitored postoperatively (Fig. 5B). Patients detected with CH‐related mutations from plasma were longitudinally monitored after the primary tumor resection to assess the clinical significance of excluding CH‐related mutations in the application of ctDNA for disease and treatment monitoring. The misclassification of CH‐related mutations as tumor‐derived mutations leading to incorrect clinical interpretation as the presence of residual disease was observed in three patients. In patient 2, one CH‐related mutation was detected in the preoperative plasma sample, tumor tissue, and PBCs. This mutation was subsequently detected in all three postoperative plasma samples with a similar VAF despite no signs of metastasis (Fig. 6A). Similarly, the CH‐related mutation detected in the preoperative plasma sample in patient 27 was also consistently detected in two postoperative plasma samples (Fig. 6B). In this case, the ctDNA mutation (*TP53*‐Y220C), which was present in both the tumor tissue and the preoperative plasma sample, but not in PBCs, was no longer detectable in the postoperative samples. Importantly, five mutations were detected in the preoperative plasma sample from patient 44, who underwent adjuvant chemotherapy after tumor resection. Three of these five mutations were also detected in the tumor tissues, and one (*TP53*‐C176Y) was detected in the corresponding PBCs. This CH‐related mutation was consistently detected in the two postoperative plasma samples, while other tumor‐derived mutations were not detected after adjuvant chemotherapy (Fig. 6C).

## 4 Discussion

As the use of NGS technologies for cfDNA continues to expand in clinical settings, accurate identification of ctDNA mutations is critically important to enhance the application of this technology in early cancer screening, personalized cancer treatment decision, monitoring minimum residual disease, and monitoring responses to treatments. Recently, a large number of studies have been conducted to evaluate the robustness of cfDNA in clinical applications by assessing the mutations in concordance between tumor tissue DNA and plasma cfDNA. However, diverse concordance rates have been reported across studies as a result of nonstandardized sample processing methodologies, assay platforms, and study designs (Bolivar *et al*., 2019; Chae *et al*., 2016; Wyatt *et al*., 2017). In addition, the complex composition of plasma cfDNA, which contains a mixture of germline DNA, DNA from clonally expanded hematopoietic cells in addition to DNA from malignant cells, further increases the complication in the interpretation of mutations detected in plasma samples (Bauml and Levy, 2018; Wan *et al*., 2017). A better understanding of potential sources of discrepancies among the studies is essential to standardize the methodologies for accurate identification of ctDNA mutations in plasma samples and to implement the clinical application of liquid biopsy. In this study, we have demonstrated that CH‐related mutations can be detected in both tumor tissues and plasma samples, which can be mistakenly inferred as tumor‐derived mutations leading to inaccurate interpretation of the blood‐based liquid biopsy results.

In this study, we have applied the same ultradeep targeted NGS platform across all three sources of DNA to detect mutations with very low VAF in the corresponding tissue, plasma, and PBC samples. This allowed us to identify CH‐related mutations even from tumor tissues which are often missed in standard sequencing analysis of tumor tissues. For instance, in the study conducted by Hu *et al*. (2018), the tumor tissues were sequenced to a mean target depth of 187x and all the CH‐related mutations detected from plasma samples were not identified in the tumor tissue samples. The insufficient sequencing depth may account for the lower detection rate of CH‐related mutation from tumor tissues and plasma compared to our study cohort (Hu *et al*., 2018).

Similar to previous studies, the VAF of the CH‐related mutations detected in the tumor tissues in our study was significantly lower than the cancer cell‐derived mutations, possibly due to the lower number of white blood cells compared to the tumor cells in the tissues (Coombs *et al*., 2018; Kleppe *et al*., 2015; Li *et al*., 2017; Ptashkin *et al*., 2018). In contrast, CH‐related mutations and tumor‐derived mutations were detected at similar VAF in plasma which makes differentiating CH‐related mutations from tumor‐derived mutations challenging. Current approaches to exclude non‐tumor‐derived mutations, such as germline variants, from ctDNA are by evaluating the VAF threshold and cross‐referencing to existing human genome databases (Xu, 2018). In addition, majority of CH‐related mutations are classified as tumor‐derived somatic mutations, which further complicates the curation of variants. For instance, *TP53*‐R293C was observed as both tumor‐derived mutations and CH‐related mutations in two different patients. Therefore, solely modifying variant calling pipelines by comparing the VAF or filtering out specific mutations would unlikely be effective to exclude CH‐related mutations from plasma samples. Sequencing of corresponding patient PBCs and plasma samples is essential to accurately determine tumor‐derived mutations from liquid biopsy.

In our study, 23 (36%) of the 64 mutations that were identified from plasma were undetected in the tumor tissues. In addition, similar discordance rates of 28–84% have been reported in several previous studies (Barata *et al*., 2017; Chae *et al*., 2016; Li *et al*., 2019). A study conducted by Rothwell *et al*. (2019) demonstrated that NGS of cfDNA and patient's corresponding tumor tissues from 39 patients with advanced‐stage solid tumor revealed up to 30% of mutations identified from plasma samples were not detected in tumor tissues. Results from our study confirmed that the misclassification of CH‐related mutation as tumor‐derived mutations in plasma samples partially contributes to the observed discordance of mutations detected between plasma and tumor tissue samples. However, several other factors have also been suggested to contribute to the discordance between ctDNA and tissue, for example, intratumoral heterogeneity, intertumoral heterogeneity, and potential assay errors (Merker *et al*., 2018). Previous studies reported that approximately 10–11% of advanced colorectal cancers showed intratumoral heterogeneity of *KRAS* or *BRAF* mutations where mutations were identified in only one of the multiple formalin‐fixed, paraffin‐embedded tumor blocks (Nelson *et al*., 2018; Richman *et al*., 2011). In our study, two pieces of tumor tissues were available from nine patients and one discordance in the *KRAS* mutation was observed in one out of the nine (11%) patients (data not shown). Furthermore, in two patients, in whom no mutation was identified in their tumor tissues, we detected four mutations that were exclusively presented in the plasma samples, further illustrating the potential utility of liquid biopsy to detect intratumor heterogeneity. Although our findings suggest intratumor heterogeneity and misclassification of CH‐related mutations can contribute to the discrepancies observed, further studies are required to deepen our understanding of the biological sources of cfDNA and other factors that may contribute to the discordant mutations detected in plasma and tumor tissue samples.

The detection of CH‐related mutations in plasma liquid biopsy could greatly affect the decision‐making process in clinical settings. As seen in our study, the detection of CH‐related mutations in plasma samples could be incorrectly interpreted as an indication of residual disease after tumor resection (Fig. 6A,B). The consistent detection of CH‐related mutations in plasma samples could also be inappropriately inferred as disease progression or treatment ineffectiveness (Fig. 6C). Furthermore, CH‐related mutations detected in plasma samples may have a more significant clinical implication in screening of early‐stage cancers as they can cause false‐positive judgment. It has also been reported that CH‐related mutations are detected in approximately 10% of nonmalignant individuals (Young *et al*., 2017) and the prevalence of these mutations increases with age and smoking (Steensma, 2018). The targeted NGS panel used in this study, Oncomine Pan‐Cancer, covers the most commonly detected mutations from solid tumors; however, it does not cover the genes that are commonly associated with hematopoietic stem cells, such as *DNMT3A*, *TET2,* and *ASXL1* (Ptashkin *et al*., 2018; Razavi *et al*., 2019). The utilization of larger gene panels that also cover genes which are associated with hematological malignancies may detect more CH‐related mutations, resulting in a higher prevalence of CH detected from cfDNA. This also emphasizes the importance of sequencing paired plasma–buffy coat using the same targeted panel to avoid selecting CH‐related mutation as disease monitoring markers. Misinterpretation of these CH‐related mutations as ctDNA mutations may lead to unreliable diagnosis for early cancer screening, as well as misguided targeted therapy, resulting in poor clinical management.

There are several technical limitations to this study. The PBCs obtained for this study are collected from the buffy coat after the removal of plasma. The collected buffy coat is treated and washed by buffer to remove the residual plasma and prevent the contamination of cfDNA. Although the multiple washing steps should remove majority of the residual cfDNA, the possibility of minute ctDNA contamination present in the DNA extracted from PBCs cannot be entirely excluded. Similarly, the contribution of circulating tumor cells to buffy coat may also lead to tumor‐specific mutation contamination in the sequencing analysis of PBCs. However, it has been reported previously that circulating tumor cells shed from primary or metastatic foci are extremely rare with an average of 1–5 cells circulating in one milliliter of blood (Yoon *et al*., 2019). This is contrasted to the presence of 8 × 10^6^ of white blood cells in 1 mL of blood under normal circumstances, which suggests the extracted DNA contributed by circulating tumor cells to the overall DNA extracted from buffy coat would likely to be minute and insignificant to the mutations detected from PBCs. Future advancement in sampling processing and DNA extraction is required to further eliminate tumor‐derived mutation contamination in PBC sequencing. The variant calling pipeline and variant filtering used in this study were error‐corrected and evaluated using healthy control plasma samples to reduce technical noise. The quality score threshold, minimum allele frequency, minimum variant score, and strand bias for each mutation were adjusted accordingly. Despite the use of multiple strategies to alleviate sequencing artifacts, the precise contribution of background sequencing noise to the detected mutations at or near the limit of detection of the assay cannot be completely ruled out. The accumulation of larger data sets together with machine learning could allow further improvement in the accuracy of mutation detection in future studies.

## 5 Conclusions

Deep NGS of patient‐paired cfDNA, PBCs, and tumor tissue performed in our study demonstrated that a substantial fraction of mutations detected from plasma had features consistent with CH‐related mutations that may lead to incorrect interpretation of liquid biopsy results. Based on our results and previously published data, paired plasma–PBC sequencing should be performed as the standard practice for NGS genomic analysis of cfDNA to avoid misinterpretation of results, leading to misguided clinical management for cancer patients.

## Conflict of interest

Yoon Ming Chin and Rie Hayashi were reported as employees of Cancer Precision Medicine Inc, Japan. Kazuma Kiyotani and Siew‐Kee Low reported consulting or advisory roles with Cancer Precision Medicine Inc, Japan. Yusuke Nakamura reported consulting and advisory roles with OncoTherapy Science, Inc, Japan.

## Author contributions

HTC, SN, YN, and S‐KL conceived and designed the study. SN, YF, and MU carried out patient recruitment. HTC, SN, YMC, and S‐KL performed acquisition, analysis, or interpretation of data. HTC, SN, and S‐KL drafted the manuscript. HTC, SN, YMC, YN, and S‐KL contributed to critical revision of the manuscript for important intellectual content. HTC, MO, RH, and KK provided technical and material support. SN, YF, MU, YN, and S‐KL were the supervisors for this project. All authors have read and approved the final manuscript.

## Supporting information


**Fig. S1.** Distribution of mutations detected from tumor tissues and plasma.
**Fig. S2.** Frequency of CH‐related mutations detected from PBCs by age increases with patient age.
**Table S1.** Clinical and pathological characteristics of the study cohort.
**Table S2.** Sequencing coverage and performance parameters.
**Table S3.** Summary of all mutations detected from tumor tissues.
**Table S4.** Summary of all mutations detected from pre‐operative plasma.
**Data S1.** Supplementary Methods.
