# Supplementary material for: Clinical significance of clonal hematopoiesis in the interpretation of blood liquid biopsy
Source: Mol Oncol. 2020 Jun 8;14(8):1719–30. doi: 10.1002/1878-0261.12727 (PMC7400786; doi:10.1002/1878-0261.12727)
Supplement: Supplementary file 1 — Fig. S1. Distribution of mutations detected from tumor tissues and plasma. Fig. S2. Frequency of CH‐related mutations detected from PBCs by age increases with patient age. Table S1. Clinical and pathological characteristics of the study cohort. Table S2. Sequencing coverage and performance parameters. Table S3. Summary of all mutations detected from tumor tissues. Table S4. Summary of all mutations detected from pre‐operative plasma. Data S1. Supplementary Methods. [file MOL2-14-1719-s001.docx]

**Clinical significance of clonal hematopoiesis in the interpretation of blood liquid biopsy**

Online Supplementary Information

**Supplementary Table 1: Clinical and pathological characteristics of the study cohort**

| **Total number of patients** | | 38 |  |
| --- | --- | --- | --- |
| **Median Age (years)** |  | 65.4 | (42-88) |
| **Gender** |  |  |  |
|  | Male | 24 | (63%) |
|  | Female | 14 | (37%) |
| **Primary site of disease** | |  |  |
|  | Cecum | 6 | (16%) |
|  | Ascending | 4 | (10.5%) |
|  | Transverse | 4 | (10.5%) |
|  | Descending | 2 | (5%) |
|  | Sigmoid | 5 | (13%) |
|  | Rectum | 17 | (45%) |
| **Stage** |  |  |  |
|  | I | 7 | (18%) |
|  | II | 13 | (34%) |
|  | III | 15 | (40%) |
|  | IV | 3 | (8%) |
| **Depth** |  |  |  |
|  | T1 | 4 | (11%) |
|  | T2 | 4 | (11%) |
|  | T3 | 20 | (53%) |
|  | T4 | 10 | (26%) |
| **Metastasis** |  |  |  |
|  | 0 | 42 | (93%) |
|  | 1 | 3 | (7%) |
| **Lymph nodes invasion** | |  |  |
|  | N0 | 21 | (55%) |
|  | N1 | 10 | (26%) |
|  | N2 | 6 | (16%) |
|  | N3 | 1 | (3%) |

| **Sample** | **Input (ng)** | **Median read coverage** | **Median molecular coverage** | **Ratio (Mean read coverage/ median molecular coverage)** |
| --- | --- | --- | --- | --- |
| 2 | 13 | 38736.5 | 3031 | 12.78 |
| 3 | 13 | 43400 | 3643 | 11.91 |
| 4 | 15 | 43301.5 | 3771 | 11.48 |
| 5 | 11 | 29773.5 | 2553 | 11.66 |
| 6 | 10 | 33765.5 | 2193 | 15.40 |
| 7 | 14 | 44745 | 3291.5 | 13.59 |
| 8 | 19 | 45727.5 | 4617.5 | 9.90 |
| 10 | 10 | 44264 | 3344 | 13.24 |
| 11 | 20 | 60267 | 4791 | 12.58 |
| 12 | 10 | 55806 | 3320.5 | 16.81 |
| 13 | 20 | 47419 | 5251 | 9.03 |
| 14 | 11 | 38415.5 | 3204 | 11.99 |
| 17 | 17 | 44544 | 3693 | 12.06 |
| 18 | 17 | 51131 | 4444.5 | 11.50 |
| 19 | 17 | 47350 | 4265 | 11.10 |
| 20 | 20 | 48193 | 4682 | 10.29 |
| 21 | 13 | 45112 | 3390 | 13.31 |
| 22 | 12 | 51581.5 | 3534 | 14.60 |
| 23 | 19 | 54573.5 | 4988.5 | 10.94 |
| 24 | 16 | 47991 | 4157.5 | 11.54 |
| 25 | 17 | 47191 | 3686.5 | 12.80 |
| 26 | 12 | 41794.5 | 3361.5 | 12.43 |
| 27 | 14 | 42610 | 4087.5 | 10.42 |
| 29 | 19 | 49318 | 3920.5 | 12.58 |
| 30 | 20 | 56801 | 4182 | 13.58 |
| 31 | 20 | 66249 | 4872.5 | 13.60 |
| 32 | 12 | 39214 | 3346.5 | 11.72 |
| 33 | 20 | 58129 | 5763.5 | 10.09 |
| 35 | 20 | 51589 | 4910 | 10.51 |
| 36 | 18 | 39312.5 | 4470.5 | 8.79 |
| 37 | 9 | 22897 | 2015 | 11.36 |
| 38 | 20 | 56957.5 | 4119.5 | 13.83 |
| 39 | 20 | 46678 | 4394.5 | 10.62 |
| 40 | 15 | 58325.5 | 4585.5 | 12.72 |
| 41 | 20 | 43576.5 | 4569 | 9.54 |
| 42 | 20 | 47985.5 | 5277 | 9.09 |
| 43 | 20 | 42656 | 3407.5 | 12.52 |
| 44 | 11 | 34349.5 | 2832 | 12.13 |

**Supplementary Table 2: Sequencing coverage and performance parameters**

**Supplementary Table 3: Summary of all mutations detected from tumor tissues. CH-related mutations are highlighted in the table.**

| **Sample** | **Locus** | **Genotype** | **Type** | **Genes** | **Amino Acid  Change** | **Mol  Depth** | **Mol  Counts** | **Mol Freq %** |
| --- | --- | --- | --- | --- | --- | --- | --- | --- |
| C02 | chr17:7577568 | C/G | SNV | TP53 | p.C238S | 1374 | 5 | 0.36 |
| C03 | chr5:112175390 | C/T | SNV | APC | p.Q1367* | 521 | 132 | 25.34 |
| C03 | chr17:7577124 | C/T | SNV | TP53 | p.V272M | 1097 | 530 | 48.31 |
| C05 | chr17:7577570 | C/T | SNV | TP53 | p.M237I | 1566 | 260 | 16.60 |
| C07 | chr3:178936091 | G/A | SNV | PIK3CA | p.E545K | 2503 | 154 | 6.15 |
| C07 | chr7:55221830 | G/A | SNV | EGFR | p.V292M | 3444 | 1286 | 37.34 |
| C07 | chr17:7578383 | AGCAGCGCTCATGGTGGGGGC/AGCAGCGCT CATGGTGGGGGA | SNV | TP53 | p.C176F | 2466 | 331 | 13.42 |
| C07 | chr20:57484421 | G/A | SNV | GNAS | p.R201H | 3172 | 393 | 12.39 |
| C08 | chr3:178936091 | G/A | SNV | PIK3CA | p.E545K | 2592 | 613 | 23.65 |
| C08 | chr12:25398279 | CGC/CGT | SNV | KRAS | p.G13D | 3164 | 1524 | 48.17 |
| C10 | chr3:178936092 | A/C | SNV | PIK3CA | p.E545A | 1175 | 501 | 42.64 |
| C10 | chr12:25398283 | ACC/AAC | SNV | KRAS | p.G12V | 3054 | 1757 | 57.53 |
| C10 | chr17:7577543 | C/T | SNV | TP53 | p.M246I | 1252 | 481 | 38.42 |
| C12 | chr20:57484420 | C/T | SNV | GNAS | p.R201C | 3808 | 2 | 0.05 |
| C12 | chr17:7577538 | C/T | SNV | TP53 | p.R248Q | 1047 | 418 | 39.92 |
| C12 | chr17:7578463 | C/T | SNV | TP53 | p.R156H | 1350 | 2 | 0.15 |
| C13 | chr12:56482341 | G/T | SNV | ERBB3 | p.D297Y | 2765 | 391 | 14.14 |
| C13 | chr17:7577121 | G/A | SNV | TP53 | p.R273C | 1510 | 321 | 21.26 |
| C13 | chr11:534288 | C/T | SNV | KRAS | p.G12D | 3032 | 22 | 0.73 |
| C14 | chr1:11184573 | G/T | SNV | MTOR | p.S2215Y | 1574 | 1352 | 85.90 |
| C14 | chr12:25398279 | CGC/CGT | SNV | KRAS | p.G13D | 2527 | 1538 | 60.86 |
| C17 | chr17:7578413 | C/T | SNV | TP53 | p.V173M | 2123 | 246 | 11.59 |
| C18 | chr7:140453134 | TCA/TCT | SNV | BRAF | p.V600E | 260 | 96 | 36.92 |
| C19 | chr17:7577538 | C/T | SNV | TP53 | p.R248Q | 1049 | 463 | 44.14 |
| **Sample** | **Locus** | **Genotype** | **Type** | **Genes** | **Amino Acid  Change** | **Mol  Depth** | **Mol  Counts** | **Mol Freq %** |
| C20 | chr7:140453134 | TCA/TCT | SNV | BRAF | p.V600E | 203 | 5 | 2.46 |
| C20 | chr17:7578272 | G/A | SNV | TP53 | p.H193Y | 1458 | 33 | 2.26 |
| C21 | chr3:178936091 | G/A | SNV | PIK3CA | p.E545K | 1251 | 379 | 30.30 |
| C21 | chr5:112175674 | AAAG/AA | INDEL | APC | p.S1465fs | 324 | 256 | 79.01 |
| C21 | chr12:25398283 | ACC/AAC | SNV | KRAS | p.G12V | 3345 | 1576 | 47.12 |
| C22 | chr17:7577511 | A/G | SNV | TP53 | p.L257P | 855 | 710 | 83.04 |
| C23 | chr3:178936092 | A/G | SNV | PIK3CA | p.E545G | 1452 | 403 | 27.75 |
| C23 | chr12:25398283 | ACC/ATC | SNV | KRAS | p.G12D | 2505 | 513 | 20.48 |
| C23 | chr17:7577121 | G/A | SNV | TP53 | p.R273C | 915 | 373 | 40.77 |
| C23 | chr17:7577094 | G/A | SNV | TP53 | p.R282W | 1865 | 2 | 0.11 |
| C24 | chr7:140453134 | TCA/TCT | SNV | BRAF | p.V600E | 414 | 27 | 6.52 |
| C24 | chr17:7578475 | G/A | SNV | TP53 | p.P152L | 2024 | 93 | 4.59 |
| C25 | chr5:112175162 | C/T | SNV | APC | p.Q1291* | 1067 | 466 | 43.67 |
| C25 | chr12:25378647 | T/A | SNV | KRAS | p.K117N | 2987 | 2134 | 71.44 |
| C25 | chr17:7578536 | T/C | SNV | TP53 | p.K132E | 1484 | 1247 | 84.03 |
| C25 | chr17:7578457 | C/T | SNV | TP53 | p.R158H | 1719 | 6 | 0.35 |
| C25 | chr3:178936094 | C/A | SNV | PIK3CA | p.Q546K | 1233 | 4 | 0.32 |
| C26 | chr12:25398283 | ACC/ATC | SNV | KRAS | p.G12D | 3128 | 1168 | 37.34 |
| C26 | chr17:7577538 | C/T | SNV | TP53 | p.R248Q | 1025 | 603 | 58.83 |
| C27 | chr17:7578190 | T/C | SNV | TP53 | p.Y220C | 1803 | 331 | 18.36 |
| C27 | chr17:7577570 | C/T | SNV | TP53 | p.M237I | 1277 | 1 | 0.08 |
| C27 | chr3:178952085 | A/G | SNV | PIK3CA | p.H1047R | 764 | 10 | 1.45 |
| C29 | chr12:25398279 | CGC/CGT | SNV | KRAS | p.G13D | 2914 | 2536 | 87.03 |
| C29 | chr17:7578383 | AGCAGCGCTCATGGTGGGGGCAGC/AGCAGC | INDEL | TP53 | p.P177_C182del | 914 | 625 | 68.38 |
| C29 | chr17:7578406 | C/T | SNV | TP53 | p.R175H | 737 | 471 | 63.91 |
| C29 | chr20:57484421 | G/A | SNV | GNAS | p.R201H | 3764 | 1192 | 31.67 |
| C30 | chr5:112174631 | C/T | SNV | APC | p.R1114* | 1878 | 830 | 44.20 |
| **Sample** | **Locus** | **Genotype** | **Type** | **Genes** | **Amino Acid  Change** | **Mol  Depth** | **Mol  Counts** | **Mol Freq %** |
| C30 | chr17:7578492 | C/T | SNV | TP53 | p.W146* | 1232 | 851 | 69.07 |
| C31 | chr12:25398283 | ACC/ACG | SNV | KRAS | p.G12R | 2958 | 792 | 26.77 |
| C31 | chr17:7577094 | G/A | SNV | TP53 | p.R282W | 1575 | 965 | 61.27 |
| C33 | chr5:112173917 | C/T | SNV | APC | p.R876* | 2317 | 1005 | 43.38 |
| C33 | chr5:112175639 | C/T | SNV | APC | p.R1450* | 795 | 256 | 32.20 |
| C33 | chr12:25398283 | ACC/ATC | SNV | KRAS | p.G12D | 2507 | 835 | 33.31 |
| C33 | chr17:7577538 | C/T | SNV | TP53 | p.R248Q | 1563 | 10 | 0.64 |
| C35 | chr12:25378562 | C/T | SNV | KRAS | p.A146T | 2183 | 1247 | 57.12 |
| C35 | chr17:7577547 | C/T | SNV | TP53 | p.G245D | 1623 | 1289 | 79.42 |
| C36 | chr17:7577094 | G/A | SNV | TP53 | p.R282W | 883 | 642 | 72.71 |
| C37 | chr3:178936082 | G/A | SNV | PIK3CA | p.E542K | 1553 | 179 | 11.53 |
| C37 | chr17:7577539 | G/A | SNV | TP53 | p.R248W | 1486 | 443 | 29.81 |
| C37 | chr17:7577121 | G/A | SNV | TP53 | p.R273C | 1833 | 7 | 0.38 |
| C38 | chr17:7578410 | T/A | SNV | TP53 | p.R174W | 2281 | 848 | 37.18 |
| C39 | chr12:25398283 | ACC/AAC | SNV | KRAS | p.G12V | 2924 | 1285 | 43.95 |
| C40 | chr12:25398283 | ACC/ATC | SNV | KRAS | p.G12D | 3476 | 1274 | 36.65 |
| C40 | chr17:7577022 | G/A | SNV | TP53 | p.R306* | 1179 | 896 | 76.00 |
| C41 | chr4:153247289 | G/A | SNV | FBXW7 | p.R505C | 2676 | 635 | 23.73 |
| C41 | chr5:112175216 | G/T | SNV | APC | p.E1309* | 396 | 273 | 68.94 |
| C43 | chr17:7577538 | C/T | SNV | TP53 | p.R248Q | 1133 | 304 | 26.83 |
| C44 | chr7:140453134 | TCA/TCT | SNV | BRAF | p.V600E | 111 | 6 | 5.41 |
| C44 | chr17:7578263 | G/A | SNV | TP53 | p.R196* | 2234 | 255 | 11.41 |
| C44 | chr17:7578403 | C/T | SNV | TP53 | p.C176Y | 2245 | 5 | 0.22 |

**Supplementary Table 4: Summary of all mutations detected from pre-operative plasma. CH-related mutations are highlighted in the table.**

| **Sample** | **Locus** | **Genotype** | **Type** | **Genes** | **Amino Acid Change** | **Mol Depth** | **Mol Counts** | **Mol Freq %** |
| --- | --- | --- | --- | --- | --- | --- | --- | --- |
| C02 | chr17:7577568 | C/G | SNV | TP53 | p.C238S | 2588 | 41 | 1.58 |
| C05 | chr17:7577570 | C/T | SNV | TP53 | p.M237I | 1956 | 1 | 0.05 |
| C06 | chr12:25398283 | ACC/ATC | SNV | KRAS | p.G12D | 2120 | 5 | 0.24 |
| C06 | chr17:7577535 | C/A | SNV | TP53 | p.R249M | 1758 | 3 | 0.17 |
| C06 | chr17:7578458 | G/A | SNV | TP53 | p.R158C | 1742 | 25 | 1.44 |
| C07 | chr17:7577535 | C/A | SNV | TP53 | p.R249M | 2424 | 3 | 0.12 |
| C07 | chr7:55221830 | G/A | SNV | EGFR | p.V292M | 3406 | 1 | 0.03 |
| C08 | chr5:112175639 | C/T | SNV | APC | p.R1450* | 3684 | 12 | 0.33 |
| C08 | chr12:25398279 | CGC/CGT | SNV | KRAS | p.G13D | 4283 | 17 | 0.40 |
| C08 | chr17:7577535 | C/A | SNV | TP53 | p.R249M | 3848 | 5 | 0.13 |
| C08 | chr17:7578508 | C/T | SNV | TP53 | p.C141Y | 3721 | 3 | 0.08 |
| C10 | chr17:7577538 | C/T | SNV | TP53 | p.R248Q | 2597 | 4 | 0.15 |
| C10 | chr3:178936092 | A/C | SNV | PIK3CA | p.E545A | 3521 | 2 | 0.06 |
| C11 | chr17:7577120 | C/T | SNV | TP53 | p.R273H | 3235 | 6 | 0.19 |
| C11 | chr17:7578518 | C/T | SNV | TP53 | p.A138T | 4214 | 4 | 0.09 |
| C12 | chr3:178952072 | A/G | SNV | PIK3CA | p.M1043V | 2872 | 2 | 0.07 |
| C12 | chr17:7577538 | C/T | SNV | TP53 | p.R248Q | 2552 | 32 | 1.25 |
| C12 | chr17:7578463 | C/T | SNV | TP53 | p.R156H | 3199 | 3 | 0.09 |
| C12 | chr20:57484420 | C/T | SNV | GNAS | p.R201C | 4113 | 13 | 0.32 |
| C13 | chr5:112175639 | C/T | SNV | APC | p.R1450* | 4013 | 11 | 0.27 |
| C13 | chr12:25398283 | ACC/ATC | SNV | KRAS | p.G12D | 4769 | 8 | 0.17 |
| C13 | chr17:7577535 | C/A | SNV | TP53 | p.R249M | 3775 | 11 | 0.29 |
| C13 | chr17:7577121 | G/A | SNV | TP53 | p.R273C | 3351 | 5 | 0.15 |
| C14 | chr1:11184573 | G/T | SNV | MTOR | p.S2215Y | 2931 | 2 | 0.07 |
| **Sample** | **Locus** | **Genotype** | **Type** | **Genes** | **Amino Acid Change** | **Mol Depth** | **Mol Counts** | **Mol Freq %** |
| C14 | chr17:7578536 | T/G | SNV | TP53 | p.K132Q | 2833 | 5 | 0.18 |
| C18 | chr2:42522656 - chr2:29446394 |  | FUSION | EML4(13) - ALK(20) |  |  | 21 |  |
| C19 | chr17:7577538 | C/T | SNV | TP53 | p.R248Q | 3298 | 3 | 0.09 |
| C20 | chr1:11184589 | C/T | SNV | MTOR | p.A2210T | 4270 | 3 | 0.07 |
| C20 | chr7:140453134 | TCA/TCT | SNV | BRAF | p.V600E | 4498 | 24 | 0.53 |
| C20 | chr17:7578272 | G/A | SNV | TP53 | p.H193Y | 5079 | 25 | 0.49 |
| C21 | chr12:25398283 | ACC/AAC | SNV | KRAS | p.G12V | 2864 | 1 | 0.03 |
| C22 | chr17:7577511 | A/G | SNV | TP53 | p.L257P | 2431 | 13 | 0.53 |
| C23 | chr7:140453134 | TCA/TCT | SNV | BRAF | p.V600E | 4167 | 9 | 0.22 |
| C23 | chr17:7577094 | G/A | SNV | TP53 | p.R282W | 3454 | 7 | 0.20 |
| C24 | chr7:140453134 | TCA/TCT | SNV | BRAF | p.V600E | 2973 | 2 | 0.07 |
| C25 | chr3:178936094 | C/A | SNV | PIK3CA | p.Q546K | 3839 | 8 | 0.21 |
| C25 | chr5:112175162 | C/T | SNV | APC | p.Q1291* | 3059 | 6 | 0.20 |
| C25 | chr17:7577094 | G/C | SNV | TP53 | p.R282G | 1947 | 2 | 0.10 |
| C25 | chr17:7578457 | C/T | SNV | TP53 | p.R158H | 3249 | 5 | 0.15 |
| C25 | chr17:7579377 | G/A | SNV | TP53 | p.Q104* | 2600 | 4 | 0.15 |
| C25 | chr12:25378647 | T/A | SNV | KRAS | p.K117N | 4432 | 2 | 0.05 |
| C26 | chr17:7578263 | G/A | SNV | TP53 | p.R196* | 3634 | 10 | 0.28 |
| C27 | chr17:7577570 | C/T | SNV | TP53 | p.M237I | 3221 | 11 | 0.34 |
| C27 | chr17:7578190 | T/C | SNV | TP53 | p.Y220C | 4370 | 2 | 0.05 |
| C30 | chr5:112174631 | C/T | SNV | APC | p.R1114* | 3997 | 6 | 0.15 |
| C30 | chr17:7578492 | C/T | SNV | TP53 | p.W146* | 3448 | 13 | 0.38 |
| C31 | chr12:25398283 | ACC/ACG | SNV | KRAS | p.G12R | 4861 | 1 | 0.02 |
| C33 | chr17:7577539 | G/A | SNV | TP53 | p.R248W | 5003 | 4 | 0.08 |
| C33 | chr5:112175639 | C/T | SNV | APC | p.R1450* | 4213 | 1 | 0.02 |
| C35 | chr12:25378562 | C/T | SNV | KRAS | p.A146T | 3561 | 21 | 0.59 |
| C35 | chr17:7577547 | C/T | SNV | TP53 | p.G245D | 4107 | 1 | 0.02 |
| **Sample** | **Locus** | **Genotype** | **Type** | **Genes** | **Amino Acid Change** | **Mol Depth** | **Mol Counts** | **Mol Freq %** |
| C36 | chr17:7577094 | G/A | SNV | TP53 | p.R282W | 2914 | 16 | 0.55 |
| C36 | chr17:7577559 | G/A | SNV | TP53 | p.S241F | 3742 | 4 | 0.11 |
| C37 | chr17:7577121 | G/A | SNV | TP53 | p.R273C | 1411 | 5 | 0.35 |
| C37 | chr17:7577539 | G/A | SNV | TP53 | p.R248W | 1714 | 4 | 0.23 |
| C39 | chr12:25398283 | ACC/AAC | SNV | KRAS | p.G12V | 4300 | 9 | 0.21 |
| C40 | chr12:25398283 | ACC/ATC | SNV | KRAS | p.G12D | 4838 | 1 | 0.02 |
| C41 | chr4:153247289 | G/A | SNV | FBXW7 | p.R505C | 4208 | 367 | 8.72 |
| C41 | chr5:112175211 | TAAAAG/TAAAAT | SNV | APC | p.E1309* | 3594 | 512 | 14.25 |
| C43 | chr17:7577538 | C/T | SNV | TP53 | p.R248Q | 2148 | 31 | 1.44 |
| C44 | chr7:55242462 | CAAGGAATTAAGAGAAGC/CAA | INDEL | EGFR | p.Glu746_Ala750del | 2643 | 5 | 0.19 |
| C44 | chr7:140453134 | TCA/TCT | SNV | BRAF | p.V600E | 2138 | 8 | 0.37 |
| C44 | chr17:7578263 | G/A | SNV | TP53 | p.R196* | 2766 | 14 | 0.51 |
| C44 | chr17:7578403 | C/T | SNV | TP53 | p.C176Y | 2945 | 3 | 0.10 |
| C44 | chr17:7578461 | C/T | SNV | TP53 | p.V157I | 2135 | 3 | 0.14 |

**Supplementary Figure 1: Distribution of mutations detected from tumor tissues and plasma**


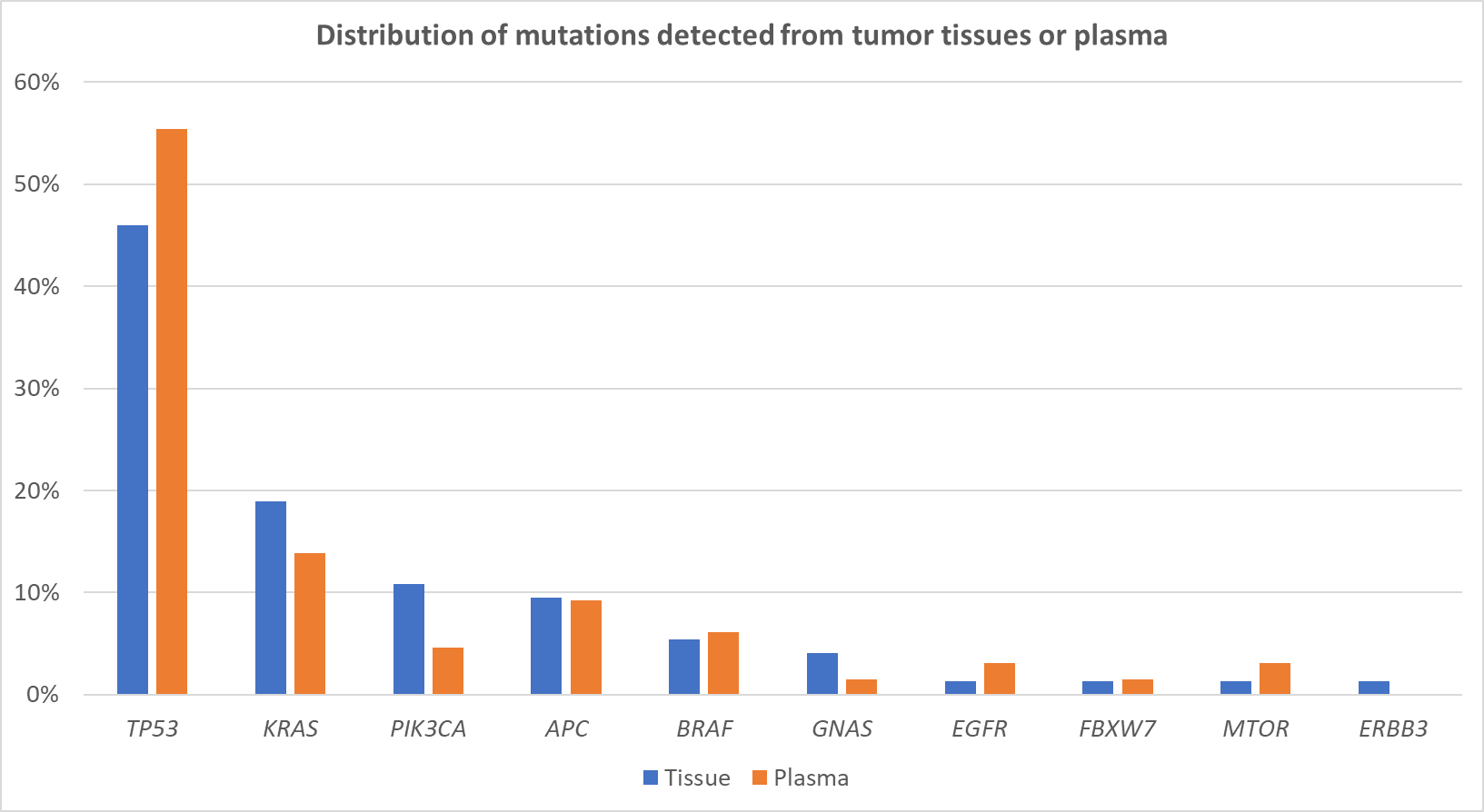


**Supplementary Figure 2: Frequency of CH-related mutations detected from PBCs by age increases with patient age.**

n= 11, 22 and 5 for age: 40-59, 60-79 and 80-89 respectively.

**Supplementary Methods**

**Sequencing data analysis software:**

Alignment of sequencing raw data: Torren Suite software v5.10.1 (Thermo Fisher Scientific)

Variant calling: Ion Reporter Software- Oncomine Tagseq Pan-Cancer Liquid Biopsy w2.1 v5.10

Annotator: Oncomine Pan-Cancer Annotations v1, using the following databases:

| Name | Version |
| --- | --- |
| 5000Exomes | 20161108 |
| Canonical RefSeq Transcripts | v83 |
| ClinVar | 20180225 |
| dbSNP | 150 |
| DGV | 20160515 |
| DrugBank | 20170911 |
| ExAC | 1 |
| Gene Ontology | 20171101 |
| Named Variants | 1 |
| Pfam | 31 |
| PhyloP Scores | 20160919 |
| RefSeq Functional Canonical Transcripts Scores | 7 |
| RefSeq GeneModel | 83 |

**Threshold for sequencing quality control metrics:**

Total coverage ≥ 20,000

Alternative allele coverage ≥ 13

Minimum mapping quality ≥ 90% of total reads

Base quality: Q20 ≥ 90% of total aligned bases

LOD ≥ 0.065%

**Definition of specific terminologies:**

Sequence depth: the number of unique reads which represents the number of times that a given nucleotide in the genome has been sequenced.

Molecular depth: The depth or number of families (sets of reads) with the same molecular barcode which represents the deduplicated reads.
